# Supplementary material for: Sleep Quality Worsens While Perceived Stress Improves in Healthcare Workers over Two Years during the COVID-19 Pandemic: Results of a Longitudinal Study
Source: Healthcare (Basel). 2022 Aug 22;10(8):1588. doi: 10.3390/healthcare10081588 (PMC9408655; doi:10.3390/healthcare10081588)
Supplement: Supplementary file 1 [file healthcare-10-01588-s001.zip › healthcare-1849904-supplementary.pdf]

Supplemental Table S1 Changes in sleep quality components scores of the study participants with difference expressed as post COVID-19 minus baseline.

Component	Baseline (T1)*	Post COVID-19 (T2)*	diff. [95% CI]**	p-value***	Cohen's d
Subjective sleep quality	1.27 ± 0.78	1.25 ± 0.97	-0.02 [-0.14 — 0.1]	0.734	0.03
Sleep latency	1.38 ± 1.05	1.42 ± 1.14	0.05 [-0.06 — 0.15]	0.364	0.08
Sleep duration	1.08 ± 1.03	1.14 ± 1.11	0.06 [-0.05 — 0.16]	0.303	0.09
Sleep efficiency	0.78 ± 1.06	0.85 ± 1	0.07 [-0.02 — 0.16]	0.141	0.12
Sleep disturbance	1.32 ± 0.59	1.26 ± 0.96	-0.06 [-0.18 — 0.07]	0.373	0.07
Use of sleep medication	0.35 ± 0.65	0.53 ± 0.71	0.18 [0.11 — 0.26]	0.001	0.4
Daytime dysfunction	1.1 ± 0.77	1.1 ± 0.89	0.01 [-0.1 — 0.12]	0.9	0.01

Notes: *Mean ± SD. ** difference (Δ) is post-baseline. ***Paired sample t-test, significant at $p < 0.05$. PSQI = Pittsburgh sleep quality index; PSS-10 = perceived stress scale, T1 is baseline = initial COVID-19 outbreak, T2 is post = after two years of the initial COVID-19 outbreak.
